# Effect of donor age and 3D-cultivation on osteogenic differentiation capacity of adipose-derived mesenchymal stem cells

**DOI:** 10.1038/s41598-020-67254-5

**Published:** 2020-06-26

**Authors:** Stephan Payr, Tim Schuseil, Marina Unger, Claudine Seeliger, Thomas Tiefenboeck, Elizabeth R. Balmayor, Martijn van Griensven

**Affiliations:** 1 Department of Experimental Trauma Surgery, Klinikum rechts der Isar, Technical University Munich, Munich, Germany; 2 University Clinic of Orthopedics and Trauma Surgery, Department of Trauma Surgery, Medical University of Vienna, Vienna, Austria; 3 Department IBE, MERLN Institute, Maastricht University, Universiteitssingel 40, 6229 ER Maastricht, the Netherlands; 4 Department cBITE, MERLN Institute, Maastricht University, Universiteitssingel 40, 6229 ER Maastricht, the Netherlands

**Keywords:** Preclinical research, Stem-cell research

## Abstract

**Background and Purpose:** Age and co-morbidities compromise healing tendencies of traumatic fractures in geriatric patients. Non-healing fractures may need regenerative medicine techniques involving autologous mesenchymal stem cells (MSCs). Donor age may affect the viability and differentiation capacity of MSCs. We investigated age-related differences in adipose-derived MSCs (AMSCs) concerning osteogenic potential in 2D and 3D cultivation. **Materials and Methods:** AMSCs were harvested from young (mean age: 37.5 ± 8.6 years) and old (mean age: 75.8 ± 9.2 years) patients. Cells were induced to osteogenic differentiation and cultivated in 2D and 3D for 14 days. Alkaline phosphatase (ALP) activity, mineralization and gene expression were investigated. **Results:** ALP activity revealed highest levels in 3D of old AMSCs after 14 days. ALP expression showed significant rises in old vs. young cells in 2D (p = 0.0024). Osteoprotegerin revealed the highest levels in old AMSCs in 2D. Highest osteocalcin levels presented in young cells compared to old cells in 2D (p = 0.0258) and young cells in 3D (p = 0.0014). **Conclusion:** 3D arrangement of old AMSCs without growth factors is not ensuring superior osteogenesis *in vitro*. AMSCs, especially cells from older patients, reveal higher osteogenic potential in 2D than in 3D. 3D arrangement favors osteogenic potential of young cells.

## Introduction

Non-healing fractures represent an increasing concern in trauma- and orthopedic surgery. The healing capacity of these fractures is often compromised by patients’ age and co-morbidities including diabetes and osteoporosis^1^. This may be partly due to age dependent differences in immune-reaction^2^. Such delayed or non-healing fractures may require specialized treatment including tissue engineering. As part of the tissue engineering approach, tissue-specific cells are needed to grow *de-novo* tissue. Because of the unique ability to differentiate into different cell phenotypes, mesenchymal stem cells (MSCs) are a promising tool for tissue engineering^3,4^. MSCs can be harvested from both autologous and allogeneic sources. Due to their low immunogenicity, allogeneic implantation has been claimed to be feasible. However, autologous MSCs present several advantages. They are not expected to elicit immune reactions or transmit diseases. Moreover, cellular expansion steps can be avoided in which the early progenitor cell population may be lost. Considering both, the use of autologous MSCs to treat non-healing fractures and the incidence of such fractures in geriatric patients, the effect of donor age on MSCs characteristics is clearly relevant. Numerous studies have investigated donor-dependent factors such as gender and body-mass- index, which might influence differentiation capacities of derived MSCs^5,6^. In particular, the effect of donor age on the MSCs cellular function has been discussed contradictorily both *in vitro* and *in vivo* studies^5–10^. There are reports showing lower^9,11,12^ equal^5,6,13^ or higher^14^ osteogenic capacity of adipose derived MSCs (AMSCs) isolated from old donors compared to those isolated from young donors. In the majority of these studies, donor age ranges from 20 to over 70 and groups were divided in decades. Most of these studies have been conducted in monolayer cultivation, which unfortunately does not match the live tissue scenario. Only few studies have been found using a 3D cultivation system to overcome the issue of cell senescence^12,15^. In addition, resulting MSCs differentiation depending on donor age has been poorly evaluated (e.g. gene expression analysis for scarce osteo-related genes, insufficient mineralization assays)^5,6,8,9,13^. Thus, only inconclusive data is available. Therefore, this study was carried out to investigate the osteogenic differentiation capacity of human AMSCs isolated from young and old donors. The cultivation system was considered as relevant. Thus, 2D and 3D cultivation were considered.

## Methods

### Materials

All reagents were purchased from Sigma-Aldrich (Munich, Germany) unless otherwise mentioned.

### Cell isolation and cultivation

Human adipose tissue samples were collected from 11 patients (n = 6 young donors: mean age was 37.5 ± 8.6 years; further referred to as *young* AMSCs; n = 5 old donors: mean age was 75.8 ± 9.2 years; further referred to as *old* AMSCs). The tissue was obtained from healthy patients undergoing abdominal, cosmetic or reconstructive surgery with written informed patient’s consent. The study was approved by the Institutional Review Board of the Technical University Munich. The study was conducted according to the Declaration of Helsinki in its latest amendment.

Adipose tissue was cut into small pieces and transferred to a falcon tube containing Dulbecco’s phosphate buffered saline (DPBS). After several washing steps, the fat pieces were incubated in an equal volume of 0.5 mg/ml collagenase type II (Biochrom, Berlin, Germany) solution at 37 °C for 30 minutes. Pre-warmed complete culture medium i.e. DMEM 4 high glucose supplemented with 10% fetal calf serum (FCS), 1% penicillin/streptomycin (P/S; PAA, Pasching, Austria) was added, mixed carefully and centrifuged at 600 g for 10 minutes. After removing the upper fat layer and the supernatant, the cell pellet was resuspended with complete DMEM. Subsequently, the cell suspension was filtered through a 70μm cell strainer (BD Falcon, NJ, USA) and plated at 3000 cells/cm^2^. Cells were cultured in monolayer until passage 3. Then young and old AMSCs were seeded either in 2D and 3D culture systems. For the 2D culture, 1×10^4^cells/cm^2^ were seeded in 24-well polystyrene plates. For 3D culture, 1.5×10^6^ cells were seeded per scaffold. A 200 μm thick polystyrene scaffold (Alvetex Scaffolds, Reinnervate, Sedgefield, England) was used (∅ 22 mm, 13 μm thin struts and pores with a mean diameter of 40 μm). The scaffolds were sterilized by 30 minutes incubation in 70% ethanol. Cells were cultured, both in 2D and 3D, using an osteogenic culture medium (i.e. DMEM low glucose supplemented with 5% FCS, 1% P/S, 10 mM β-glycerophosphate, 1.56 mM CaCl2, 100 nM dexamethasone, 0.025 M HEPES (PAA, Pasching, Austria) and 0.2 mM L-ascorbic- 2-phosphat. Medium was changed every second day. Samples were collected on days 0, 3, 7 and 14 to perform further analysis.

### Alkaline phosphatase activity

Alkaline phosphate substrate solution (i.e. 3.5 mM 4-nitrophenyl-phosphate-di-sodium- hexahydrate salt in a 0.1 mM ALP buffer (50 mM glycine, 100 mM tris-base, 2 mM MgCl2, pH 10.5)) was added to 2D and 3D cultures. After 30 minutes incubation at 37 °C, absorbance of the supernatants was measured at 405 nm. P-nitrophenol concentrations were calculated according to a standard curve and normalized to the protein content via SRB determination^16^. ALP substrate solution without cells was used as negative control.

### Alizarin red staining and quantification

Alizarin red staining was performed to evaluate mineralization of young and old AMSCs cultivated in 2D or 3D. Cells were fixed with ice-cold ethanol 96% for 30 minutes in a 24-well plate. Subsequently, 400 μl Alizarin red staining solution was added to each well for 10 minutes and washed several times. The stain was eluted using a solution of 10% hexadecylpyridiniumchloride. Absorbance was measured at 562 nm. Alizarin red concentration was calculated according to the standard curve and results were normalized to the protein content analyzed via SRB staining^16^.

### Sulforhodamine-B determination

This measurement was used to normalize Alizarin red staining and ALP activity. SRB staining solution was added to previously fixed cells and incubated for 30 minutes at RT. Subsequently, cells were washed with 1% acetic acid solution followed by 10 mM un- buffered TRIS solution and incubated 15 minutes at RT. Absorbance was measured at 565 nm and 690 nm. Total protein content was calculated by using the following equation^17^:

$${\rm{Total}}\,{\rm{protein}}[{\rm{\mu }}{\rm{g}}/{\rm{\mu }}{\rm{l}}]=({\rm{OD}}-0.\,021)/0.\,0197$$

### Total RNA isolation and reverse transcriptase polymerase chain reaction

Cell seeded scaffolds and cell monolayers were collected using TRIzol reagent. Total RNA was isolated based on phenol/chloroform method. The concentration and purity of total RNA was determined spectrophotometrically (BioPhotometer, Eppendorf, Hamburg, Germany). cDNA synthesis was performed using First strand cDNA synthesis kit (Fermentas, St. Leon- Rot, Germany) according to manufacturer’s instruction using a Master Cycler S (Eppendorf, Hamburg, Germany). The expression of ALP, osteoprotegerin (OPG), osteocalcin (OC), osteopontin (OP) and collagen type 1α1 (Col1) was determined by means of real-time quantitative reverse transcription polymerase chain reaction (RT-PCR). Amplification primers are listed in Table 1. SsoFast Eva Green Supermix (Bio-Rad Laboratories Inc., CA, USA) was used and real time PCR was carried out on a Bio-Rad CFX96 thermal cycler (Bio-Rad Laboratories Inc., CA, USA). β-tubulin was selected as a housekeeping gene. Relative quantification of gene expression was performed using the ΔΔCt method.

**Table 1:** Human primers used for the quantitative PCR evaluation. OC = osteocalcin, OP = osteopontin, ALP = alkaline phosphatase, OPG = osteoprotegerin, Col1=collagen type 1〈1, ®-Tub = ®-tubulin.

Gene	Forward primer 5ʹ 3ʹ	Reverse primer 5ʹ 3ʹ	Accession number
OC	CCAGCGGTGCAGAGTCCAGC	GACACCCTAGACCGGGCCGT	NM-199173.3
OP	CTCCATTGACTCGAACGACTC	CGTCTGTAGCATCAGGGTACTG	NM-000582
ALP	CTGGGCTCCAGGGATAAAGC	TCAGTGTCTCTTGCGCTTGG	NM-000478.4
OPG	CCGGAAACAGTGAATCAACTC	AGGTTAGCATGTCCAATGTG	NM-002546.3
COL1	AGCGGACGCTAACCCCCTCC	CAGACGGGACAGCACTCGCC	NM-000088.3
β-Tub	GAGGGCGAGGACGAGGCTTA	TCTAACAGAGGCAAAACTGAGCACC	NM-001069.2

### Statistical analysis

For statistical analysis, the two groups studied i.e. young vs. old consisted of n = 6 patients and n = 5 patients respectively (biological variability). In addition, each assay was conducted in triplicates. All the obtained values are reported as mean ± standard deviation. Statistical analysis was performed using GraphPad Prism version 6.00, (GraphPad Software, CA, USA). Non-parametric one-way Anova was performed followed by Tukey’s multiple comparison test. Statistical significance was set for p < 0.05.

## Results

### Alkaline phosphatase activity

**Figure 1:**
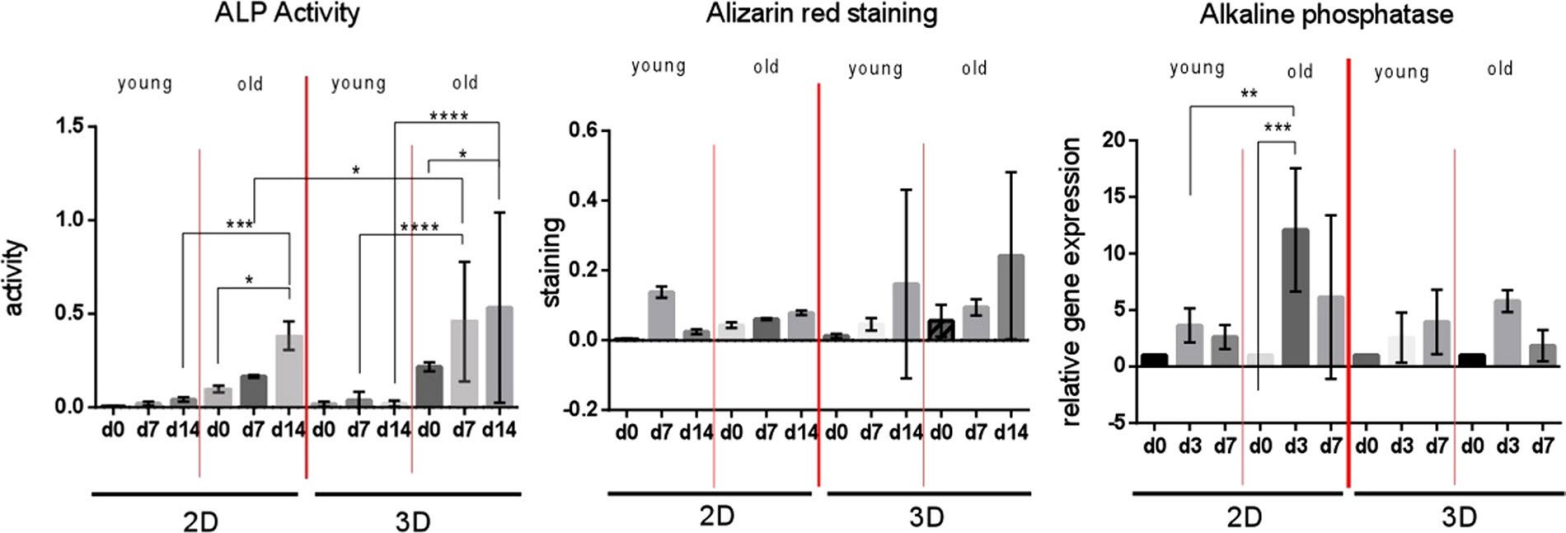
Effect of age of human AMSCs and the *in vitro* cultivation geometry on alkaline phosphatase (ALP) activity. The observation period was up to 14 days. Young cells: mean age = 37.5 years ± 8.6. Old cells: means age = 75.8 years ± 9.2. ^∗^p < 0.05, ^∗∗^p < 0.01, ^∗∗∗^p < 0.001, ^∗∗∗∗^p < 0.0001. Comparison of the mineralization potential of young versus old AMSCs after an observation period of 14 days. Both cells were cultivated in monolayer as well as 3D cultivation. Relative alkaline phosphatase (ALP) gene expression of cultivated AMSCs. Comparison of 2D versus 3D cultures and of young versus old AMSCs after an observation period of 7 days.

After 14 days in 2D culture, low ALP activity was detectable for young AMSCs while a significant increase (3.9-fold, p = 0.0494) was present in the old AMSCs (Fig. 1). When compared to young AMSCs, old AMSCs revealed higher ALP levels for all times of observation. For example, a 9-fold increase (p = 0.0007) in ALP level was obtained for old AMSCs after 14 days in monolayer culture.

In 3D culture, the ALP activity showed a similar trend for both young and old AMSCs when compared to monolayer cultivation. Young AMSCs showed low levels of ALP while old AMSCs presented significantly higher ALP levels. In the case of old AMSCs, the 3D cultivation showed a positive effect on ALP levels as slightly elevated levels were obtained for all analyzed time points. Compared to the obtained ALP base level (i.e. day 0), 2.4-fold increase was obtained for old AMSCs after 14 days in 3D culture (p = 0.0286). In summary, the 3D cultivation showed a positive effect on ALP activity of AMSCs. This can be clearly observed for old AMSCs in which a 2.75-fold increase was obtained after 7 days of culture in the 3D system (p = 0.0401). In addition, when comparing young vs. old AMSCs in 3D culture, the ALP activity levels in old cells on day 7 (12-fold; p < 0.0001) and day 14 (over 26-fold; p < 0.0001) were significantly higher than in young cells. After the completion of the 14 days observation period, the ALP activity resulted significantly higher in old AMSCs compared to young AMSCs independently of the cultivation system.

### Mineralization

Young cells in 2D culture demonstrated higher mineralization on day 7 followed by a distinct decrease on day 14 (Fig. 1). On the other hand, old AMSCs presented a continuous rise up in mineralization (1.8-fold on day 14 compared to day 0). The mineralization level in monolayer culture on day 14 is higher in old cells despite the young cells’ increase on day 7. In 3D culture, both groups show similar mineralization trends. The levels in old AMSCs were higher (but not statistically significant) on day 14 than in young cells.

### Gene expression

#### Alkaline phosphatase

In monolayer cultivation, young AMSCs presented a 3.65-fold increase of ALP gene expression on day 3, followed by a slight decrease on day 7 (Fig. 1). Old AMSCs showed a similar trend, but with a 12-fold increase on day 3 (p = 0.0001) and a reduction on day 7. Old cells showed elevated ALP expression when compared to the young AMSCs on day 3 after culture (3.3-fold, p = 0.0024). After 7 days of culture similar results were observed.

After 3D cultivation, old AMSCs showed lower ALP expression when compared to 2D but this was not statistically significant. A similar trend was observed, with a rise on day 3 followed by a distinct reduction on day 7. The maximum increase in ALP expression for these cells was detected on day 3 with a 12-fold increase for 2D vs. a 3-fold increase for 3D cultivation. Young AMSCs, on the other hand, revealed a continuous increase of ALP gene expression in 3D culture until day 7. When compared to old AMSCs, young cells showed lower ALP expression after 3 days in 3D culture. However, the expression was slightly higher for the young cells after 7 days of culture. The highest ALP expression was obtained for old AMSCs in 2D culture. To summarize, 3D cultivation did not show any beneficial effect on ALP gene expression. Moreover, AMSCs isolated from old donors showed significantly higher ALP expression when compared to the young derived ones.

### Osteoprotegerin

**Figure 2:**
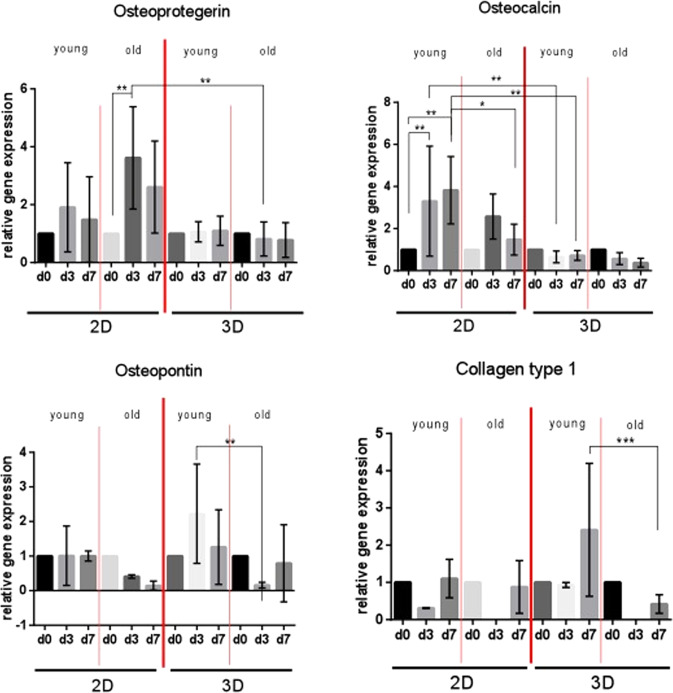
Relative gene expression of osteoprotegerin (OPG), osteoclacin (OC), osteopontin (OP) and collagen type 1 (Col1) on cultivated AMSCs for a period of up to 7 days. Comparison of 2D versus 3D cultures and of young (mean age = 37.5 years ± 8.6) versus old (75.8 years ± 9.2) cells. ^∗^p < 0.05, ^∗∗^p < 0.01.

OPG showed a similar expression pattern for the entire observation time for both, young and old cells in 2D culture (Fig. 2). The OPG expression was, however, higher in old AMSCs when compared to the young ones. In the old cells, a 3.6-fold increase (p = 0.0042) was obtained after 3 days of culture. This increase in OPG expression resulted almost twice as high for old cells when compared to the young ones after 3 days in monolayer culture (3.6 fold vs. 1.9 fold). In 3D culture, OPG expression was in general lower than after 2D cultivation for both young and old AMSCs. No significant changes in the OPG expression were detected after 3 and 7 days of 3D culture for any of the studied cells. A minimal decrease of OPG expression was revealed in old AMSCs but this was not statistically significant. In general, higher OPG expression levels were detected in the cells cultivated in monolayer compared to cells in 3D culture. Old AMSCs showed the highest OPG expression throughout the study.

### Osteocalcin

OC expression significantly increased for young AMSCs with the time of culture in 2D. A 3.3-fold and 3.8-fold increase was obtained after 3 and 7 days of culture, respectively. The increment was statistically significant for both times of observation when compared to the baseline expression level (p = 0.0093 for day 3 and p = 0.0028 for day 7) (Fig. 2). In the case of old AMSCs, they also revealed elevated OC expression on day 3, but this was followed by a distinct reduction on day 7. In addition, the OC expression was generally lower in old cells when compared to young cells in monolayer culture and for all observation times studied. At the final observation time of 7 days, the OC expression was 2.6-fold higher for young cells when compared to old cells (p = 0.0258) in 2D culture. On the other hand, when cultivated in 3D culture, young and old AMSCs demonstrated similar expression patterns of OC. In addition, the expression level of OC was significantly reduced in 3D compared to monolayer culture. Comparing the two cultivation systems for young cells, significantly higher OC expression was found in 2D culture (day 3 a 5-fold increase p = 0.0014 and day 7 a 5.3-fold increase p = 0.0027). This result was similarly observed for old cells, but to a lesser extent. In summary, a significantly higher OC expression was found in the 2D culture when compared to 3D culture for both young and old AMSCs. Young AMSCs have overall higher OC expression than old AMSCs.

### Osteopontin

Young AMSCs did not reveal any detectable expression of OP in 2D culture. However, a reduction of 86% in OP expression (i.e. related to the baseline level on day 0) was observed for old AMSCs on day 7 (Fig. 2). In 3D culture, young cells showed a boost in OP expression levels after 3 days of culture compared to day 0. This was followed by a reduction on day 7 close to the levels of the baseline value (day 0). For old AMSCs, however, a significant decrease in OP expression was obtained after 3 days of 3D cultivation. On day 7, there was a recovery to OP expression levels similar to those obtained on day 0 (baseline value, no 3D cultivation). A significantly higher OP expression was found in young cells compared to old cells. This effect was most pronounced after 3 days of cultivation in 3D culture (13.8-fold increase; p = 0.0014). On day 7, these levels are only higher by trend. In addition, 3D culture appears to stimulate the OP expression for both cell age groups studied.

### Collagen type 1α1

The expression levels of Col1 were almost identical between young and old AMSCs in 2D culture (Fig. 2). In 3D culture, a 2.4-fold increase of Col1 expression was observed in young AMSCs on day 7 compared to day 0. Old AMSCs, on the other hand, showed a reduction of the Col1 expression on day 7 when compared to day 0 (baseline expression level). When comparing the Col1 expression for both cells after 7 days of 3D cultivation, young cells expressed Col1 significantly higher than old cells (p = 0.0004). The main observation here was that 3D cultivation appeared to stimulate Col1 expression, but only when AMSCs isolated from young donors were used.

## Discussion

Decreased cell number and proliferation as well as differentiation capacity has been reported for MSCs isolated from old donors^2^. Thus, compromising MSCs implantation therapies in geriatric patients. Age-related changes of MSCs have been documented in both *in vitro* and *in vivo* studies using mostly mice and rat-derived cells^18,19^. Mueller *et al*. showed an age-related decrease on the osteogenic potential of human bone marrow-derived MCSs (BMCSs) based on a decrease of ALP activity and expression after 3 weeks^20^. This observation is not completely in line with the results obtained from our study. This study used AMSCs instead of BMSCs and the material and morphology of the scaffold used to provide the 3D cultivation was different. Within the observation period of 14 days, an age-related decrease of ALP activity was not observed; in fact, the highest levels were found in old cells cultivated in 3D scaffolds. The ALP gene expression was slightly more consistent with an age-related decline of osteogenic potential. One further detrimental age-related effect mentioned in the literature is a decrease of OPG^21^. Makhluf *et al*. described 5-fold elevated levels of OPG gene expression in MSCs isolated from patients younger than 65 years^21^. In monolayer culture, the current results showed that old AMSCs expressed OPG at higher levels than young cells. Interestingly, OPG expression levels were consistently higher in monolayer than in 3D. The expression pattern was very similar between young and old AMSCs when the 3D cultivation was performed. Thus, the age-related decline cannot be confirmed. 3D cultivation seems to inhibit OPG expression. In old cells, the gene expression patterns are very similar for ALP, OPG and OC. This is true for both cultivation systems compared in this current study. Noticeable, there is repetitively a distinct peak in gene expression on day 3 followed by a decrease on day 7. This early striking peak in gene expression may be explained considering a “cell memory” effect of old cells. Such an effect has been described by Landgraf *et al*. in human MSCs isolated from old donors^22^. The authors demonstrated that old MSCs still have a conserved suppressive effect on T-cell proliferation – like their young counterparts. The distinct decrease of expression levels following the peak may be age-related in a way that the early gene expression cannot be maintained due to cell senescence and levels sink to even lower levels than in young MSCs. One further point that demonstrated a cell memory effect is the fact that the ALP activity, which is an index for early commitment of cells to the osteoblast lineage, reveals its highest levels in old AMSCs in this current study. This effect matches the one reported by Ashton *et al*.^23^. 3D cultivation methods have been used in order to overcome a detrimental donor age-related effect on MSCs osteogenic capacity. Scaffolds used for cell cultivation contained hydroxyapatite (HA), silicon-containing compounds or inorganic compounds^24–28^. Unfortunately, no monolayer cultivation was performed using the same material surface. This is necessary in order to have a comparison based solely on the geometry effect (i.e. 2D and 3D-cultivation) rather than material composition. In our study, the same material was used for monolayer and 3D-cultivation. These current results partially confirmed the positive effect of 3D-cultivation on ALP activity and mineralization of old AMSCs. However, this advantage of 3D-cultivation to improve osteogenesis was completely absent in this current study when gene expression was analyzed by PCR for old AMSCs. The majority of the investigated osteogenic markers revealed higher gene expression levels in monolayer culture than in 3D. Further studies using HA, calcium-phosphate (CaP) ceramics or inorganic compounds achieved superior levels of osteogenic markers in 3D culture^26,27^. However, this effect seems to be more related with the material composition (i.e. HA and CaP known to be osteoconductive) than with the scaffold geometry^24,26,29^. Another study further underlined the fact that the 3D-arrangement alone is not enough to ensure superior osteogenesis^28^. The authors investigated ectopic bone formation via micropores in CaP ceramics. Data suggest that the percentage of bone volume in the scaffold’s channels was not affected by the type or size of pores, but it was mainly influenced by the surface morphology of the ceramic material^28^. In general, there are only a few investigations directly comparing 2D and 3D- cultivation models on osteogenic potential of human MSCs^24,26^. In fact, direct comparisons are difficult to perform due to the variability of 3D-systems such as biomaterial used and scaffold shape, type and morphology. In this current study, the same material in both 2D and 3D-cultivation was used. The aim was to establish a fair comparison in order to obtain relevant data in terms of osteogenic influence of monolayer or spatial cultivation of stem cells. A limitation of this current study is that the 3D-arrangement of cells *in vitro* is only mimicking, but missing real physiological *in vivo* conditions. This study analyzed the expression of five osteogenic genes in human AMSCs in order to gain as much information as possible at the gene expression level. This is in contrast to other studies mentioned in the literature in which mostly only ALP and RunX2 are assayed^20,24,25,27^. Concluding from this, this study demonstrated that 3D-arrangement of human AMSCs isolated from old donors without growth factors stimulation does not guarantee superior osteogenesis *in vitro*. Human AMSCs, especially cells from older patients, reveal higher osteogenic potential in 2D than in 3D-culture. On the other hand, *in vitro* cultivation using 3D-arrangement favors the osteogenic potential of young MSCs when compared to old cells.
