# Author Correction: Effect of donor age and 3D-cultivation on osteogenic differentiation capacity of adipose-derived mesenchymal stem cells

**DOI:** 10.1038/s41598-020-69876-1

**Published:** 2020-07-31

**Authors:** Stephan Payr, Tim Schuseil, Marina Unger, Claudine Seeliger, Thomas Tiefenboeck, Elizabeth R. Balmayor, Martijn van Griensven

**Affiliations:** 1 Department of Experimental Trauma Surgery, Klinikum rechts der Isar, Technical University Munich, Munich, Germany; 2 University Clinic of Orthopedics and Trauma Surgery, Department of Trauma Surgery, Medical University of Vienna, Vienna, Austria; 3 Department IBE, MERLN Institute, Maastricht University, Universiteitssingel 40, 6229 ER Maastricht, The Netherlands; 4 Department cBITE, MERLN Institute, Maastricht University, Universiteitssingel 40, 6229 ER Maastricht, The Netherlands

Correction to: *Scientific Reports*10.1038/s41598-020-67254-5, published online 26 June 2020

In the original version of this Article, the author Martijn van Griensven was incorrectly indexed. This error has now been corrected.

